# Long-term outcomes of benznidazole treatment in chronic Chagas disease: A 27-year cohort study of parasitological cure and death in the Jequitinhonha Valley, Brazil

**DOI:** 10.1371/journal.pntd.0013619

**Published:** 2025-11-20

**Authors:** Júlio César Santoro de Oliveira Assis, Lourena Tomazelli Suave, Gabriela Roberta Ramos Fernandes, Girley Francisco Machado-de-Assis, Helen Rodrigues Martins, Glaucia Diniz Alessio, Olindo Assis Martins-Filho, Renato Afonso Salgado, Mariângela Carneiro, Rosália Morais Torres, Marta de Lana

**Affiliations:** 1 Programa de Pós-graduação em Ciências Biológicas, Núcleo de Pesquisa em Ciências Biológicas, Universidade Federal de Ouro Preto, Ouro Preto, Minas Gerais, Brazil; 2 Programa de Pós-Graduação em Ciências Farmacêuticas, Escola de Farmácia, Universidade Federal de Ouro Preto, Ouro Preto, Minas Gerais, Brazil; 3 Departamento de Ciências Básicas da Vida, Universidade Federal de Juiz de Fora, Campus Governador Valadares, Governador Valadares, Minas Gerais, Brazil; 4 Universidade Federal dos Vales do Jequitinhonha e Mucuri, Diamantina, Minas Gerais, Brazil; 5 Grupo Integrado de Pesquisas em Biomarcadores, Instituto René Rachou/FIOCRUZ-Minas, Belo Horizonte, Minas Gerais, Brazil; 6 Hospital Nossa Senhora das Graças, Sete Lagoas, Minas Gerais, Brazil; 7 Departamento de Parasitologia, Instituto de Ciências Biológicas, Universidade Federal de Minas Gerais, Belo Horizonte, Minas Gerais, Brazil; 8 Faculdade de Medicina, Universidade Federal de Minas Gerais, Belo Horizonte, Minas Gerais, Brazil; Centro de Pesquisa Gonçalo Moniz-FIOCRUZ/BA, BRAZIL

## Abstract

**Background:**

Chagas disease (CD) is a neglected tropical infection prevalent in rural areas of Latin America. The etiological treatment is indicated for patients with both acute and chronic phases of CD, except in the most advanced clinical stage of the disease. However, there are still several divergences regarding the benefits of etiological treatment in chronic CD as far as the parasitological cure and survival/mortality outcomes. The present study aimed at verifying the impact of benznidazole (BZ) treatment on parasitological cure and death of patients with chronic CD at 9, 13, and 27 years post-treatment.

**Objective:**

This study aimed to verify the impact of BZ-treatment on parasitological cure and death of patients with chronic CD at 9, 13, and 27-year follow-up.

**Methods:**

A historical and prospective cohort of 42 patients with chronic CD, categorized as BZ-treated (BZ-T, n = 21) and Not-treated (NT, n = 21), were evaluated after 9, 13, and 27 years of follow-up, using parasitological tests (Hemoculture-HC, polymerase chain reaction-PCR, and quantitative real-time PCR-qPCR), conventional-CS (ELISA), and non-conventional-NCS (Chagas-Flow ATE) serology, employing three distinct cure criteria, one classical and two alternative. Survival analysis for death was determined by the Kaplan-Meier method.

**Results:**

Parasitological data (HC, PCR and qPCR) were negative in all patients (BZ-T and NT) at 27-year follow-up. The CS was negative in 75% and 23.1% of patients from BZ-T and NT, respectively. NCS had a higher negative rate (95%) than CS (23.1%) in BZ-T. The classic cure criterion demonstrated that 75% of BZ-T achieved a successful therapeutic outcome. Moreover, the use of the second and third alternative cure criteria revealed a higher proportion of cure in BZ-T (90%). The overall mortality over 27-year follow-up was 4.8% in BZ-T and 38.1% in NT. Kaplan-Meier survival curves for survival estimated 95% and 40% for BZ-T and NT patient groups.

**Conclusions:**

The overall analysis demonstrated that BZ-T chronic CD patients yielded higher parasitological cure rates as well as increased survival over a 27-year follow-up. The Chagas-Flow ATE proved to be a valuable tool for monitoring therapeutic response and, together with parasitological and molecular parasitological methods, provided a more accurate cure criterion.

## Author summary

Chagas disease is a common parasitic disease that occurs mainly in poor regions of countries with low development rates of Latin America, including Brazil. According to the World Health Organization, it is estimated that between 6 and 7 million people are infected worldwide, with 10,000 deaths per year in historically endemic regions, such as Mexico, Central, and South America. Due to the human migratory process, CD has spread to other previously unaffected regions, such as Europe, Japan, Australia, Canada, and the United States. Specific treatment with the antiparasitic drug benznidazole is prescribed for patients infected in the acute and chronic phases. However, the studies demonstrating benefits, cure, and survival post-treatment are discordant. We periodically monitored 42 patients with CD, including: BZ-treated (n = 21) and Not-treated (n = 21) for 27-year follow-up. We analyzed the presence of the infection with three parasitological and two serological examinations. The global analysis, considering the average amongst the different cure criteria (1^st^ = 75%; 2^nd^ = 90%; 3^rd^ = 90%), revealed parasitological cure in more than 85% of patients in BZ-T and no cure in the NT group. Furthermore, the overall survival was superior in BZ-T (95.0%) as compared to NT (40.0%). The mortality was 4.8% in treated and 38.1% in Not-treated patients. Thus, our study demonstrated that long-term follow-up of BZ-treatment brought about several benefits regarding cure rates and survival, highlighting the relevance of treating patients at chronic phase of CD.

## Introduction

Chagas disease (CD), also known as American trypanosomiasis [1,2], is a tropical vector-borne infection caused by the protozoa parasite *Trypanosoma cruzi*. It is a neglected disease, originally described as a public health issue predominant in impoverished rural areas of South and Central America [3]. However, human migration from endemic areas has contributed to the global spread of this disease, especially to United States and Europe [4].

The clinical course of CD usually comprises an acute phase followed by a chronic phase, with a marked contrast between the low incidence of acute cases and the high prevalence of chronic cases [5]. The acute phase is often asymptomatic or oligosymptomatic, presenting a range of nonspecific manifestations such as fever, lymphnode hypertrophy, hepatosplenomegaly, edema, myocarditis, among others [6,7]. The chronic phase of CD is characterized by low or subpatent parasitemia, a result of the ongoing action of the humoral and cellular immune responses. This phase is classified into different clinical forms: indeterminate, cardiac, digestive or mixed (cardiodigestive) [1,5]. Most patients in the chronic phase remain asymptomatic (indeterminate form). However, around 20–40% of cases, progress to clinically evident cardiac and/or digestive manifestations over time [1,8]. The evolution of CD from the acute to the chronic phase significantly affects patients’ health status and their quality of life, making the chronic phase a notable cause of both morbidity and mortality in CD [9].

According to the second Brazilian Consensus on Chagas Disease of 2015 [10], in chronic phase, the treatment with benznidazole (BZ) is recommended for patients in the indeterminate form, as well as those with mild cardiac and/or digestive involvement, to delay and/or reduce the disease progression to more severe clinical forms [3,11]. A broader guide has been recommended by PAHO (2018) [12]. In Brazil, the treatment is administered orally at a prolonged schedule of 5–10 mg/kg/day, for 60 consecutive days [12,13]. The etiological treatment reaches high cure rates in the acute (70–90%) and early chronic infections (less than 16-years of infection), but the parasitological cure rates decline in late chronic infections [14–16]. Despite the indications for treatment, there are still many controversies among physicians and researchers about the effectiveness of BZ in the late chronic phase, as many treated asymptomatic patients progress to the symptomatic chronic phase of the disease [17,18].

Several studies have demonstrated over time that treatment with BZ showed low or no efficacy when carried out in the chronic phase, with active infection and/or electrocardiographic changes, in addition to the death outcome, when compared to the not-treated patients in a long-time follow-up [17–19].

The controversies about the cure rates achieved may reflect the use of different therapeutic schemes, diversity between the methodologies and cure criteria used, genetic differences between the parasites, time of infection and/or follow-up, age of patients and possible operational difficulties in post-treatment follow-up of CD [8,14,20,21]. Furthermore, the possibility of autoimmune events playing a role in the pathogenesis of the disease led several researchers to the conclusion that treatment of advanced stage CD was unnecessary [22]. Therefore, it is necessary to carry out new studies with long-term follow-up of treated patients of different geographical regions and consequently infected with distinct *T. cruzi* DTUs, which could provide the scientific and clinical community with more consolidated data that demonstrate the cure rates in patients at the late chronic phase of CD.

One important and controversial aspect of human treatment is the cure criterion [7,23]. Particularly in the late chronic infection, the time necessary for the detection of the parasitological cure, taking into account the classic cure criterion [7], which considers as cured those patients with negative parasitological and molecular parasitological tests, as well as the conventional serological tests [7]. However, accomplishing this criterion requires more than 20–25 year follow-up [24], due to the later seroreversion of the conventional serology (CS), with positive serological status not necessarily associated with active infection [25,26], but with several other factors [27]. However, several methodologies for assessing the effectiveness of the treatment exist and have brought new achievements, revealing early the cure [25,28]. Among these, we may cite the techniques of the non-conventional serology (NCS), such as the investigation of lytic antibodies Co [25,27,28] or their analogs anti-live trypomastigotes antibodies [23,29,30] including the detection of anti-live amastigote & trypomastigote and fixed epimastigote antibodies by flow cytometry (Chagas-Flow ATE) of several modalities [26]. Numerous immunological and biochemical or metabolic biomarkers of cure in Chagas disease, which are not focus of this study, exist [31–33]. In addition, the use of molecular parasitological techniques based on polymerase chain reaction (PCR) of higher sensitivity and specificity, particularly the real-time PCR [34], has brought gains and is being used in the monitoring of treatment activity with more accuracy in the detection of parasite DNA [35–38]. The interpretation of qPCR results in treatment assessment is that positive results indicate a present infection, and negative ones are particularly useful for indicating therapeutic action, but not a cure precisely [39].

Given this scenario, our group previously developed a study in the municipalities of Berilo and José Gonçalves de Minas, Jequitinhonha Valley, Minas Gerais, a region of intense CD transmission in the preceding decades, some years after the implementation of the epidemiological surveillance [40]. So, a cohort of patients with chronic Chagas disease treated with BZ in 1997 was evaluated with parasitological and serological monitoring after 9 and 13-year follow-up [41,42].

To further evaluate the long-term outcomes of BZ treatment in chronic CD, we have monitored the therapeutic cure throughout a 27-year follow-up, using three distinct cure criteria that employ a set of laboratory tests with different principles, natures, and sensitivities, alongside death analysis, allowing a comprehensive analysis of treatment outcomes over nearly three decades.

## Materials and methods

### Ethics statement

The study protocol was submitted and approved by the Ethics Committee for Research Involving Human of the René Rachou Institute – FIOCRUZ-Minas (CAAE: 83247817.7.0000.5091). All participants signed the informed consent form before inclusion. The experimental procedures were conducted according to the ethical standard principles outlined in the Helsinki Declaration.

### Study design

**Fig 1:**
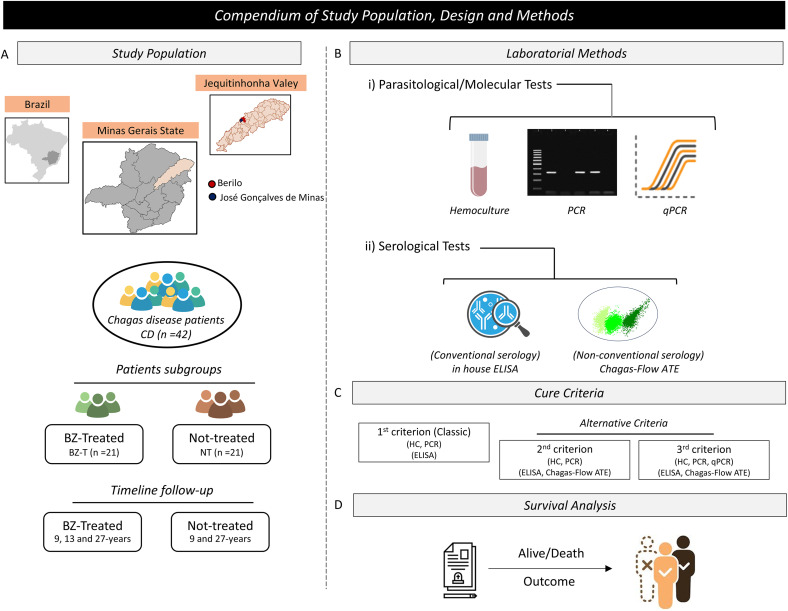
Flowchart of study population and design. (A) Study population providing the geographical location of study municipality and the patients`groups. Map of Brazil highlighting the state of Minas Gerais in gray and map of Minas Gerais State highlighting the Jequitinhonha Valley, MG, Brazil, a region of intense CD transmission in the past decades. The maps were designed by the authors using the Clip Studio Paint Ver. 3 (CELSYS, Inc, Tokyo, Japan). The base layer of the map: https://www.ibge.gov.br/geociencias/downloads-geociencias. (B) Laboratorial methods used for patients monitoring, comprising Parasitological/Molecular and Serological tests. (C) Cure Criteria employed for patients monitoring, including classic and alternative criteria. (D) Analysis of patients` survival.

The present investigation comprises a historical and prospective cohort study of chronic CD patients diagnosed with *T. cruzi* infection, throughout 27 years after BZ-treatment (BZ-T) as compared to not treated (NT) patients. All participants were born or residents in Berilo municipality, Jequitinhonha Valley, MG, Brazil, a region of intense CD transmission in the previous decades, with a large number of patients in the chronic phase of CD (Fig 1), now free of vectorial transmission of the disease [43].

### Patients

This original cohort consisted of 29 patients treated with BZ (BZ-Treated), who were matched for age (mean age 3 years’ range) and sex with 29 patients with CD who were not treated (NT). These patients were evaluated thereafter at 9 [41] and 13 years [42] later. Between the first and third evaluation, 8 (27%) treated patients were lost to follow-up.

Forty-two patients with chronic CD were enrolled in the present investigation, and divided into two groups, referred as: i) BZ-treated group (BZ-T, n = 21) patients who received standard BZ-treatment in 1996, before the second Brazilian Consensus on Chagas Disease [10] and WHO guidelines [12]. This group comprised 7 males and 14 females, mean age of 29.6 years at the time of treatment, monitored at 9, 13 and 27-year follow-up (patients were enrolled in 1997 and followed up to 2024); ii) Not-treated group (NT, n = 21) had a mean age of 32.6 years at the time of diagnosis and was reassessed 9 and 27 years after initial diagnosis (patients were enrolled in 1993 and followed up to 2024). Demographic characteristics of the study patients are presented in Table 1.

**Table 1:** Baseline features of the study of patients with chronic Chagas disease categorized as BZ-treated and NT.

Variable	Total (n = 42)	BZ-T (n = 21)	NT (n = 21)	p value
**Sex**				
Males	14 (33.3)	7 (50.0)	7 (50.0)	
Females	28 (66.7)	14 (50.0)	14 (50.0)	0.500
**Age at diagnosis (years)**				
Mean ± SD	31.1 ± 6.6	29.6 ± 5.8	32.6 ± 7.1	0.978
Median (IQR)	30.5 (7.5)	29.0 (7.5)	32.0 (9.0)	1.000
Maximum	50	41	50	
Minimum	14	14	18	
**Follow-up (years) mean ± SD**	25.5 ± 4.6	27.0 ± 0.3	24.1 ± 6.3	
**Locality (at baseline)**				
Rural	32 (76.2)	13 (61.9)	19 (90.5)	
Urban	10 (23.8)	8 (38.1)	2 (9.5)	0.165

SD: standard deviation; IQR: interquartile range.

The treatment was carried out in 1997 and consisted of the administration of the nitroderivative benznidazole (N-benzyl-2-nitroimidazole acetamide) from Roche, following all the recommendations of the Brazilian Ministry of Health. Patients received an extended therapeutic regimen of 5–10 mg/kg/day, administered orally for 40–60 consecutive days, under medical supervision, with biweekly control of leukocytes count and surveillance for potential side effects. Treatment confirmation was verified through review of medical prescriptions and/or patient’s interviews.

### Parasitological method - Hemoculture (HC)

The parasitological examination HC followed the methodology [44]. Briefly, 30 mL of heparinized venous blood was collected from each study participant, centrifuged at 3.000 rpm for 10 min and the plasma was discarded. The resulting pellet was resuspended in 15 mL of Liver Infusion Tryptose (LIT) medium and centrifuged under the same conditions. After removing the supernatant, the pellet was resuspended in 15 mL of LIT, distributed into 3 tubes, maintained at 28ºC, homogenized every 48 hours, and examined at regular intervals of 30 days for 120 days.

### Molecular-Parasitological test - Polymerase chain reaction (PCR)

PCR reactions were performed following the methodology [45]. and modified. Initially, a volume of 5 mL of venous blood was collected from study participants and mixed with an equal volume of 6 M guanidine HCl and 0.2 M EDTA, pH 8.0, as described [46]. DNA was extracted using the Blood & amp; Tissue DNA Purification Kit (Ludwig, Cat. DNK0702, Lot. 343143A), following the manufacturer’s recommendations. PCR amplifications were performed using a 9 µL reaction mixture containing 10 mM Tris-HCl (pH 9.0), 0.1% Triton X-100 (Invitrogen São Paulo, SP, Brazil), 75 mM KCl (Invitrogen São Paulo), 3.5 mM MgCl2 (Invitrogen São Paulo), 0.2 mM of each deoxynucleotide (dATP, dCTP, dGTP, dTTP; Sigma, St. Louis, MO, USA), 0.5 U of Platinum Taq DNA polymerase (Invitrogen São Paulo) and #121 forward [6.5 pmol/5’ AAATAATGTACGGGTGAGATGCATGA3’] and #122 reverse [6.5 pmol/5’ GGTTCGATTGGGGTTGGTGTAATATA3’] (Invitrogen, São Paulo, Brazil) (GENBANK: AB038240.1). Sterile water (Milli-Q) was added to bring the final reaction volume to 15 μL. Two microliters of the extracted DNA sample, containing 30 ng were added to the reaction mixture. The amplification control of the reaction was carried out in parallel to the amplification using the primers PCO3 (ACACAAACTGTGTTCACTAGC) and PCO4 (CAACTTCATCCACGTTCACC) that amplify the constitutive human beta-globin gene. PCR reactions were carried out on an automatic thermocycler (Applied Biosystems Veriti 96 Well Thermal Cycler, California, USA), as described [45]. Primers #121 and #122 anneal in the conserved micro-regions of the k-DNA minicircle, for amplification of fragments of approximately 330 base pairs (bp). The visualization of the amplified products was carried out in an electrophoresis device using a 1% agarose gel, stained with the DNA intercalator GelRed and using a 100 bp molecular weight marker (QX SizeMarker Qiagen), according to the manufacturer’s instructions. Negative internal control (sterile water) and a reagent control (mixer) were included in all experimental batches. All reactions were performed in duplicates, using negative and positive samples as reference controls.

### Molecular-Parasitological test - Quantitative real time PCR (qPCR)

Quantitative analysis of *T. cruzi* DNA was carried out using the real time qPCR as [47]. Standard curve was generated from the amplification of serial dilutions (1:10) of a parasite suspension starting from 1.0 x 10^8^ epimastigotes of *T. cruzi* Y strain, theoretically equivalent to 1.0 x 10^-1^ parasitic cells. The SDS 7500 software (Applied Biosystems) was used to calculate the linear regression coefficient (R^2^) and the Slope. The qPCR amplifications were performed from 30 ng of genomic DNA extracted from participants, together with 5 μL of the SYBR Green fluorescent dye from the GoTaq qPCR Master Mix kit (Promega) and 10 μM of each primer # 121 forward (5’ AAATAATGTACGGGTGAGATGCATGA3’) and #122 reverse (5’ GGTTCGATTGGGGTTGGTGTAATATA3’) (Invitrogen, São Paulo, Brazil) with target a region of *T. cruzi* k-DNA. The final reaction volume was adjusted to 10 μL, according to the manufacturer’s recommendations. Reactions were performed in duplicates and included wells with positive control (DNA extracted from the epimastigote forms of *T. cruzi* Y strain) and negative control (sterile water and without the presence of DNA), with the respective primers and the SYBR Green. Amplifications were performed using the ABI Prism 7500 Sequence Detection System thermocycler (Applied Biosystems, USA) in a 3-step cycling program: (i) 95ºC for 2 minutes for initial denaturation; (ii) 40 cycles of 95ºC for 15 seconds and 60ºC for 1 minute; (iii) a dissociation curve step with denaturation at 95ºC for 15 seconds, annealing at 60ºC for 1 minute, a second denaturation at 95ºC for 30 seconds, and cooling at 60ºC for 15 seconds.

### Conventional serological test - Enzyme-linked immunosorbent assay (ELISA)

In-house ELISA was performed to detect anti-*T. cruzi* immunoglobulin G (IgG) levels according to [48]., modified by [49]. The epimastigotes soluble antigen form *T. cruzi* Y strain, isolated in the exponential phase of growth in LIT media was used as target antigens at a concentration of 4.5 μg/mL. Briefly, serum samples (at 1:80 dilution) were incubated in 96-well plates, pre-coated with *T. cruzi* antigen, followed by addition of pre-diluted peroxidase-conjugated anti-human IgG (BethylLaboratories, Montgomery, USA). The IgG reactivity was measured on a microplate reader (BIO RAD, Model 3550), using a 490 nm filter. The cut-off value was calculated for each plate as the average absorbance of 10 negative control serum samples plus 2 standard deviations [49]. Data was expressed as optical densities (OD) and further reported as reactivity index (Sample OD/cut-off OD) to classify the results as negative (<1) or positive (>1). Serological monitoring was performed at distinct time points (9, 13 and 27-years) for BZ-T group and (9 and 27-years) for the NT group.

### Non-conventional serological test - Flow cytometry antibody analysis (Chagas-Flow ATE)

Non-conventional serological test (Chagas-Flow ATE) was performed at distinct time points follow-up. Chagas-Flow ATE comprises the analysis of anti-live-amastigote & trypomastigote and fixed epimastigote antibodies in serum samples previously described [26]., using the *T. cruzi* CL strain as target antigen. Briefly, in 96-well “U” bottom plates, serum samples (diluted from 1/250–1/32.000) were incubated with the parasite suspension at 37ºC for 30 minutes, followed by addition of second step reagents (biotinylated anti-human IgG1 antibody plus phycoerythrin-conjugated streptavidin-SAPE) to quantify the IgG1 reactivity. The parasite suspension was fixed with flow cytometry paraformaldehyde solution. Samples were run in flow cytometer (FACScalibur (BD Bioscience, San Diego, CA, USA) and the FlowJo software (TreeStar, San Diego, CA, USA) used for data analysis. The IgG1 reactivity was expressed as the Percentage of Positive Fluorescent Parasites (PPFP), determined over the limit of PPFP<2% set for the second step reagent internal control. The IgG1 reactivity to live-amastigote antigen at serum dilution 1:1,000 was used for monitoring BZ-T and NT patients at distinct follow-up time points, considering PPFP = 40% to classify the results as negative (<40%) or positive (>40%).

### Cure interpretation

Laboratorial cure criteria were applied considering the results of parasitological/molecular methods (HC, PCR and qPCR) together with conventional (ELISA) and nonconventional (Chagas-Flow ATE) serological tests. Patients presenting any positive parasitological and/or molecular parasitological examination were considered not cured, regardless of the results of the serological tests.

Three distinct cure criteria were established, as follows: first criterion (classical cure criterion), second and third criterion. The first cure criterion comprises negative parasitological and molecular parasitological tests (HC and PCR), as well as negative conventional serological tests (ELISA) [7]. The second cure criterion encompasses negative parasitological and molecular parasitological tests (HC and PCR), as well as negative or positive conventional serological tests (ELISA), and negative non-conventional serological tests (Chagas-Flow ATE) [26]. The third cure criterion consists of negative results in all parasitological and molecular parasitological tests (HC, PCR, and qPCR), negative non-conventional serological tests (Chagas-Flow ATE), and negative or positive conventional serological tests (ELISA).

### Survival analysis

Death was the outcome assessed in the survival analysis and was evaluated in BZ-T and NT groups at 27-year follow-up. Mortality data was obtained from medical records database (Mortality Information System/MIS), managed by the Center for Strategic Information on Health Surveillance (DATASUS), at the Health Department of the Berilo Municipality of, Minas Gerais. This database includes the underlying cause of all deaths occurring in the State of Minas Gerais and other locations in Brazil.

Cumulative mortality (expressed by number of patients progressed to death divided by the number of patients during the follow-up by 100) and mortality rate (expressed as the number of patients progressed to death divided by the person-time at risk during the follow-up time by 1,000) were described.

The survival probability was calculated by assembling the Kaplan-Meier estimate curves to compare time to death between BZ-T and NT throughout the 27-year follow-up. Sex (male and female) and age groups (41–56 and 57–68 years) was also compared by Kaplan-Meir estimate curves. The survival probability curves were compared using the log-rank test.

The Cox proportion hazard model to assess the effect of the BZ-treatment on death were used to estimate hazard ratios (HR) with confidence intervals (95% CI). Bivariate Cox analysis for risk of death was performed for treatment, sex and age. The final model for death of patients with chronic CD, categorized as BZ-treated and NT upon 27-year follow-up, was adjusted by age and sex.

## Results

### Demographic and epidemiological data

The demographic and epidemiological data of participants identified at the beginning of this study and stratified by BZ-treated (BZ-T) and Not-treated (NT), are presented in Table 1 (see Materials and Methods).

The majority of patients included in the study were female (66.7%), from rural areas (76.2%), and the mean age was 29.6 ± 5.8 (BZ-T) and 32.6 ± 7.1 (NT) years. The mean follow-up time post-treatment or diagnosis was 26.5-years.

### Parasitological analysis

#### Hemoculture (HC).

**Fig 2:**
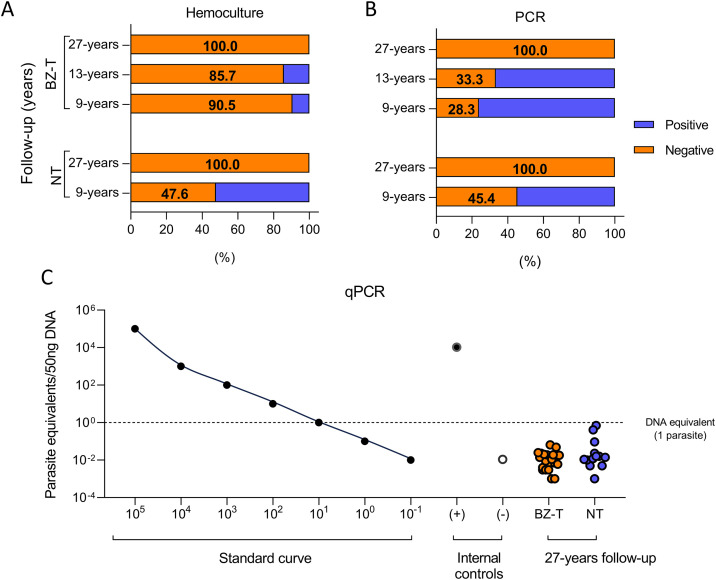
Parasitological and molecular parasitological evaluation by hemoculture (A), PCR (B), and qPCR (C) of patients with chronic CD categorized as BZ-treated and NT.

In the BZ-T group, HC was negative in 90.5% (19/21) and 85.7% (18/21) of participants at 9 and 13-year follow-up, and in 100% (20/20) of participants at 27-years (Table 2 and Fig 2).

**Table 2:** Follow-up of benznidazole treated chronic Chagas disease patients using different laboratorial methods for cure assessment.

ID	Timeline follow-up (years)															
	Parasitological/Molecular methods							Serological methods						27-year follow-up		
								CS			NCS			Cure criteria		
	Hemoculture			PCR			qPCR	ELISA			Chagas-Flow ATE					
	9^yrs^	13 ^yrs^	27 ^yrs^	9 ^yrs^	13 ^yrs^	27 ^yrs^	27 ^yrs^	9 ^yrs^	13 ^yrs^	27 ^yrs^	9 ^yrs^	13 ^yrs^	27 ^yrs^	1^st^	2^nd^	3^rd^
**447**	P	N	N	P	P	N	N	P	P	P	P	P	N	Not cured	Cured	Cured
**543**	P	P	*	N	P	*	*	P	P	*	P	*	*	*	*	*
**772**	N	N	N	P	P	N	N	P	P	P	P	P	N	Not cured	Cured	Cured
**1007**	N	P	N	N	N	N	N	P	P	N	P	P	N	Cured	Cured	Cured
**1091**	N	N	N	N	P	N	N	P	P	N	P	P	N	Cured	Cured	Cured
**1092**	N	N	N	P	P	N	N	P	P	P	P	P	N	Not cured	Cured	Cured
**1095**	N	N	N	N	N	N	N	P	P	N	P	P	N	Cured	Cured	Cured
**1096**	N	N	N	N	P	N	N	P	P	N	P	P	N	Cured	Cured	Cured
**1097**	N	N	N	P	N	N	N	P	P	P	P	P	P	Not cured	Not Cured	Not cured
**1098**	N	P	N	P	N	N	N	P	P	N	P	P	N	Cured	Cured	Cured
**1100**	N	N	N	P	P	N	N	P	P	P	P	P	P	Not cured	Not cured	Not cured
**1102**	N	N	N	P	P	N	N	P	P	N	P	P	N	Cured	Cured	Cured
**1103**	N	N	N	P	P	N	N	P	P	N	P	P	N	Cured	Cured	Cured
**1104**	N	N	N	P	P	N	N	P	P	N	P	P	N	Cured	Cured	Cured
**1105**	N	N	N	N	N	N	N	P	P	N	P	P	N	Cured	Cured	Cured
**1108**	N	N	N	P	N	N	N	P	P	N	P	P	N	Cured	Cured	Cured
**1109**	N	N	N	P	N	N	N	P	P	N	P	P	N	Cured	Cured	Cured
**1110**	N	N	N	P	P	N	N	P	P	N	P	P	N	Cured	Cured	Cured
**1111**	N	N	N	P	P	N	N	P	P	N	P	P	N	Cured	Cured	Cured
**1112**	N	N	N	P	P	N	N	P	P	N	P	P	N	Cured	Cured	Cured
**1113**	N	N	N	P	P	N	N	P	P	N	P	P	N	Cured	Cured	Cured
**N or Cured (%)**	**90.5**	**85.7**	**100.0**	**28.6**	**33.3**	**100.0**	**100.0**	**0.0**	**0**	**75.0**	**0.0**	**0.0**	**90.0**	**75.0**	**90.0**	**90.0**

ID = Identification number; N = Negative result; P = Positive result; CS = Conventional serology; C = Cured; NCS = Non-conventional serology; * = Death.

In the NT group, HC was negative in 47.6% of patients (10/21) at 9-year post-diagnosis, progressively increasing to 100% (13/13) at 27-year follow-up (Table 3 and Fig 2).

**Table 3:** Follow-up of not treated chronic Chagas disease patients using different laboratorial methods for cure assessment.

ID	Timeline follow-up (years)											
	Parasitological/Molecular methods					Serological methods				27-year follow-up		
						CS		NCS		Cure criteria		
	Hemoculture		PCR		qPCR	ELISA		Chagas-Flow ATE				
	9^yrs^	27 ^yrs^	9 ^yrs^	27 ^yrs^	27 ^yrs^	9 ^yrs^	27 ^yrs^	9 ^yrs^	27 ^yrs^	1^st^ Classic	2^nd^	3^rd^
**125**	N	N	P	N	N	P	N	P	N	CS (NS)	CS & NCS (NS)	CS & NCS (NS)
**389**	P	N	N/A	N	N	P	P	P	P	Not cured	Not cured	Not cured
**403**	N	N	P	N	N	P	P	P	P	Not cured	Not cured	Not cured
**415**	N	N	N	N	N	P	P	P	P	Not cured	Not cured	Not cured
**438**	P	*	P	*	*	P	*	P	*	*	*	*
**441**	N	N	N	N	N	P	N	P	N	CS (NS)	CS & NCS (NS)	CS & NCS (NS)
**443**	N	N	N	N	N	P	P	P	P	Not cured	Not cured	Not cured
**493**	P	N	P	N	N	P	P	P	P	Not cured	Not cured	Not cured
**551**	N	N	N	N	N	P	P	P	P	Not cured	Not cured	Not cured
**562**	N	*	N/P	*	*	P	*	P	*	*	*	*
**640**	N	*	N	*	*	P	*	P	*	*	*	*
**798**	P	*	P	*	*	P	*	P	*	*	*	*
**830**	P	*	N/P	*	*	P	*	P	*	*	*	*
**894**	N	N	N/P	N	N	P	P	P	P	Not cured	Not cured	Not cured
**929**	P	*	N/P	*	*	P	*	P	*	*	*	*
**1365**	P	*	P	*	*	P	*	P	*	*	*	*
**1422**	P	N	N/P	N	N	P	P	P	P	Not cured	Not cured	Not cured
**2151**	N	*	N/P	*	*	P	*	P	*	*	*	*
**2438**	P	N	N/P	N	N	P	N	P	N	CS (NS)	CS & NCS (NS)	CS & NCS (NS)
**2440**	P	N	N/P	N	N	P	P	P	P	Not cured	Not cured	Not cured
**2464**	P	N	N/P	N	N	P	P	P	P	Not cured	Not cured	Not cured
**N or Cured (%)**	**47.6**	**100.0**	45.4**	**100.0**	**100.0**	**0.0**	**23.1**	**0.0**	**23.1**	**0.0**	**0.0**	**0.0**

ID = Identification number; N = Negative result; P = Positive result; N/P = not performed; C = Cured; NS = Negative serology; CS = Conventional serology; NCS = Non-conventional serology; * = Death; ** = % obtained from 10/21 patients.

#### Molecular parasitological tests (PCR and qPCR).

In the BZ-T group, the conventional PCR was negative in 28.6% (6/21) and 33.3% (7/21) of participants at 9- and 13-year post-treatment, respectively, and in 100% (20/20) of participants at 27-year follow-up (Table 2). In the NT, PCR was negative in 45.4% (5/11) of patients at 9 years post-diagnosis, increasing to 100% (13/13) at 27 years post-diagnosis (Table 3). It was not possible to perform PCR evaluation in 10/21 of the patients in the NT group after 9 years of follow-up (Table 3). Quantitative PCR (qPCR) was negative in 100.0% of the treated patients (20/20) and in 100.0% of the NT group (13/13) at 27-year post-treatment and post-diagnosis, respectively, with detected k-DNA levels equivalent to less than one *T. cruzi* parasitic cell (<50 ng of DNA) (Fig 2 and Tables 2 and 3).

#### Conventional serological test (ELISA).

**Fig 3:**
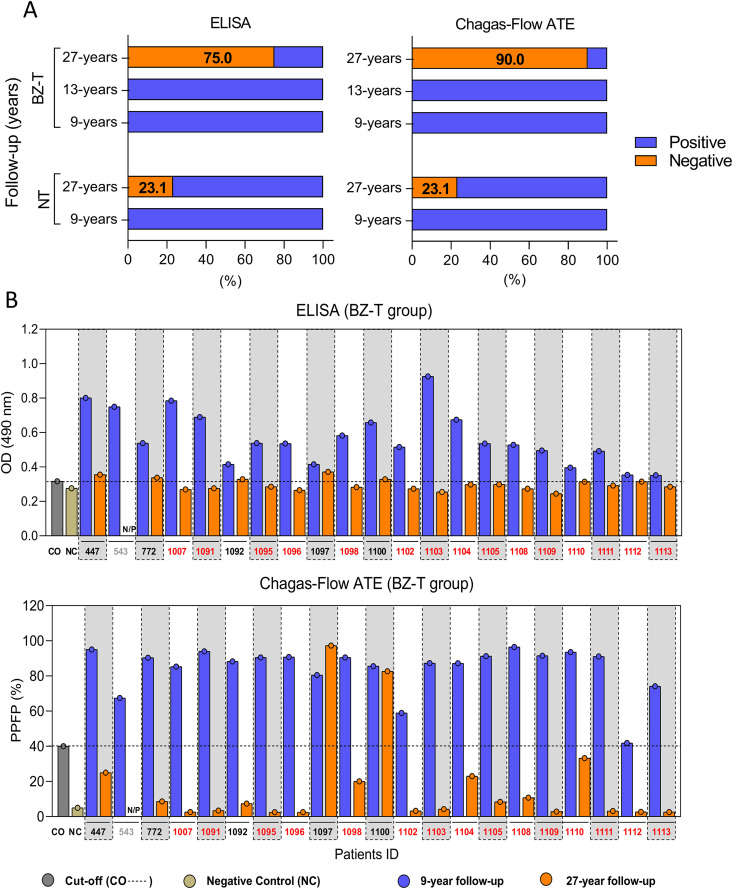
Follow-up profile of patients with chronic CD categorized as BZ-treated and NT monitored by conventional (ELISA) and non-conventional (Chagas-Flow ATE) serology. (A) Overall profile of ELISA and Chagas-Flow ATE results of BZ-T and NT groups. (B) Individual ELISA and Chagas-Flow ATE results of BZ-T group at 9-year and 27-year follow-up.

In the BZ-T group, no participant (0%, 21/21) presented negative ELISA at 9 and 13-year follow-up, while 75.0% (15/20) tested negative for ELISA at 27-year follow-up (Fig 3A and Table 2).

In the NT group, none of the patients (0%, 21/21) had negative serology at 9-year follow-up, and three patients (3/13; 23.1%) presented negative for ELISA at 27-year follow-up were (Fig 3A and Table 3).

A total of 15 patients from the BZ-T (#1007, #1091, #1095, #1096, #1098, #1102, #1103, #1104, #1105, #1108, #1109, #1110, #1111, #1112 and #1113) presented negative ELISA results at 27-year follow-up (Fig 3B, orange ID tags). Five patients (#447, #772, #1092, #1097, and #1100) remained with positive ELISA results (Fig 3B, blue ID tags). Patient #543 died before the follow-up (Fig 3B, gray ID tags).

#### Non-conventional serological test (Chagas-Flow ATE).

In Chagas-Flow ATE, the serological reactivity was considered exclusively against amastigote forms due to reactivity compatible with the ELISA test. Serological reactivity to live-trypomastigote and fixed epimastigote forms are presented in S1 and S2 Tables. In BZ-T, no patient (0%, 0/21) presented negative Chagas-Flow ATE result at 9 and 13-year follow-up. However, by 27-year follow-up, 90.0% (18/20) exhibited seronegative profiles (Fig 3A and Table 2). In the NT group, no participants were seronegative at 9-year post-diagnosis, whereas 23.1% (3/13) had negative Chagas-Flow ATE results after 27-year follow-up (Fig 3A and Table 3).

At 27-year follow-up, 18 patients from the BZ-T (#447, #772, #1007, #1091, #1092, #1095, #1096, #1098, #1102, #1103, #1104, #1105, #1108, #1109, #1110, #1111, #1112 and #1113) presented negative results for Chagas-Flow ATE (Fig 3B, orange ID tags). Two patients (#1097, #1100) presented positive results for Chagas-Flow ATE (Fig 3B, blue ID tags).

### Cure interpretation

**Fig 4:**
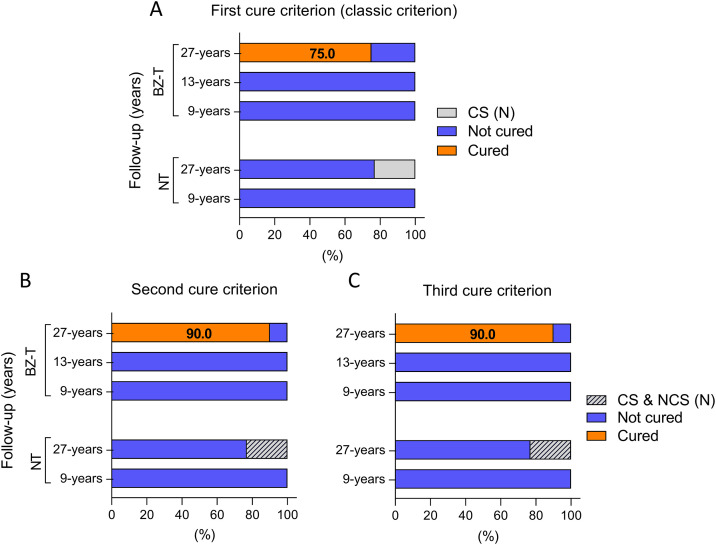
Timeline monitoring of patients with chronic CD categorized as BZ-treated and NT considering: (A) first (classic criterion); (B) second and (C) third cure criteria at distinct time points during follow-up.

i) *First cure criterion (classical criterion) -* Based on this criterion, no patient was classified as cured in BZ-T group (0/21) at 9 and 13-year follow-up nor in the NT (0/21) at 9-year follow-up (Fig 4A).

At 27-year follow-up, 75.0% (15/20) of patients in the BZ-T were considered cured, while 25.0% (5/20) remained not cured (Fig 4A and Table 2). In the NT, no patient was classified as cured 27-year follow-up (Fig 4A and Table 3). Approximately 76.9% (10/13) of the patients in this group were considered not cured, and 23.1% (3/13) serologically negative, 27-year follow-up (Fig 4A and Table 3).

ii) *Second cure criterion* - No parasitological cure was detected in BZ-T (0/21) at 9 and 13-year follow-up nor in the NT (0/21) at 9-year follow-up (Fig 4B). At 27-year follow-up, the parasitological cure was 90.0% (18/20) in BZ-T patients, with 10.0% (2/20) considered not cured (Fig 4B and Table 2). In the NT, no participants achieved parasitological cure at 27-year follow-up, meaning no patient was considered cured by this criterion (Fig 4B and Table 3). However, 23.1% (3/13) of the participants exhibited negative serology, using both ELISA and Chagas-Flow ATE method at 27-year follow-up (Fig 4B and Table 3).
iii) *Third cure criterion -* No parasitological cure was observed in the BZ-T (0/21) at 9 and 13-year follow-up nor in the NT (0/21) at 9-year follow-up, based on this criterion (Fig 4C). At 27-year follow-up, 90.0% (18/20) of patients in the BZ-T met the criterion for parasitological cure, while 10.0% (2/20) remained not cured (Fig 4C and Table 2). In the NT, no parasitological cure was observed at either 9 or 27-year follow-up. Therefore, no patient in the NT group was classified as cured by the third cure criterion (Fig 4C and Table 3). Nevertheless, 23.1% (3/13) of NT participants presented negative serology by both ELISA and Chagas-Flow ATE at 27-year follow-up (Fig 4C and Table 3).

The global analysis, considering the average amongst the different cure criteria (1^st^ = 75%; 2^nd^ = 90%; 3^rd^ = 90%), revealed parasitological cure in more than 85% of patients in BZ-T and no cure in the NT group.

Tables 4 and 5 present the laboratory findings and survival outcomes of BZ-treated and Not-treated patients in relation to the occurrence or absence of parasitological cure after 27-year follow-up.

**Table 4:** Laboratorial analysis and survival outcome in benznidazole treated chronic Chagas disease patients at 27-year follow-up.

ID	Demographic data		Laboratorial records and survival outcomes at 27-year follow-up							
			Parasitological/Molecular methods			Serological methods		Cure criteria		Survival Outcome
	Gender	Age	Hemoculture	PCR	qPCR	ELISA	Chagas-Flow ATE	1^st^ Classic	2 ^nd^ & 3^rd^	
**447**	M	50	N	N	N	P	N	Not Cured	Cured	Alive
**543**	M	68	*	*	*	*	*	*	*	Death
**772**	F	62	N	N	N	P	N	Not Cured	Cured	Alive
**1007**	F	49	N	N	N	N	N	Cured	Cured	Alive
**1091**	M	53	N	N	N	N	N	Cured	Cured	Alive
**1092**	F	56	N	N	N	P	N	Not Cured	Cured	Alive
**1095**	M	55	N	N	N	N	N	Cured	Cured	Alive
**1096**	M	58	N	N	N	N	N	Cured	Cured	Alive
**1097**	M	59	N	N	N	P	P	Not cured	Not cured	Alive
**1098**	F	63	N	N	N	N	N	Cured	Cured	Alive
**1100**	F	61	N	N	N	P	P	Not cured	Not cured	Alive
**1102**	F	56	N	N	N	N	N	Cured	Cured	Alive
**1103**	F	56	N	N	N	N	N	Cured	Cured	Alive
**1104**	F	53	N	N	N	N	N	Cured	Cured	Alive
**1105**	F	60	N	N	N	N	N	Cured	Cured	Alive
**1108**	F	53	N	N	N	N	N	Cured	Cured	Alive
**1109**	F	59	N	N	N	N	N	Cured	Cured	Alive
**1110**	F	56	N	N	N	N	N	Cured	Cured	Alive
**1111**	M	57	N	N	N	N	N	Cured	Cured	Alive
**1112**	F	63	N	N	N	N	N	Cured	Cured	Alive
**1113**	F	41	N	N	N	N	N	Cured	Cured	Alive
**N, Cured or Alive (%)**	**–**	**–**	**100.0**	**100.0**	**100.0**	**75.0**	**90.0**	**75.0**	**90.0**	**95.0**

ID = Identification number; M = Masculine; F = Feminine; N = Negative result; P = Positive result; * = Death.

**Table 5:** Laboratorial analysis and survival outcome in not treated chronic Chagas disease patients at 27-year follow-up.

Demographic data			Laboratorial records and survival outcomes at 27-year follow-up							
			Parasitological/Molecular methods			Serological methods		Cure criteria		Survival Outcome
ID	Gender	Age	Hemoculture	PCR	qPCR	ELISA	Chagas-Flow ATE	1^st^ Classic	2 ^nd^ or 3^rd^	
**125**	F	56	N	N	N	N	N	CS (NS)	CS & NCS (NS)	Alive
**389**	F	64	N	N	N	P	P	Not cured	Not cured	Alive
**403**	M	45	N	N	N	P	P	Not cured	Not cured	Alive
**415**	F	56	N	N	N	P	P	Not cured	Not cured	Alive
**438**	F	50	*	*	*	*	*	*	*	Death
**441**	F	60	N	N	N	N	N	CS (NS)	CS & NCS (NS)	Alive
**443**	M	57	N	N	N	P	P	Not cured	Not cured	Alive
**493**	M	58	N	N	N	P	P	Not cured	Not cured	Alive
**551**	F	55	N	N	N	P	P	Not cured	Not cured	Alive
**562**	M	59	*	*	*	*	*	*	*	Death
**640**	F	61	*	*	*	*	*	*	*	Death
**798**	F	56	*	*	*	*	*	*	*	Death
**830**	F	54	*	*	*	*	*	*	*	Death
**894**	F	54	N	N	N	P	P	Not cured	Not cured	Alive
**929**	M	55	*	*	*	*	*	*	*	Death
**1365**	F	63	*	*	*	*	*	*	*	Death
**1422**	F	58	N	N	N	P	P	Not cured	Not cured	Alive
**2151**	M	68	*	*	*	*	*	*	*	Death
**2438**	F	46	N	N	N	N	N	CS (NS)	CS & NCS (NS)	Alive
**2440**	M	53	N	N	N	P	P	Not cured	Not cured	Alive
**2464**	F	61	N	N	N	P	P	Not cured	Not cured	Alive
**(%) N or** **Cured or** **Alive**	**–**	**–**	**100.0**	**100.0**	**100.0**	**23.1**	**23.1**	**0.0**	**0.0**	**61,9**

ID = Identification number; M = Masculine; F = Feminine; N = Negative result; P = Positive result; CS = Conventional serology; NCS = Non-conventional serology; NS = Negative serology; * = Death.

### Survival analysis

At 27-year follow-up, overall mortality was observed in 4.8% (1/21) of patients in the BZ-T 27, compared to 38.1% (8/21) in the NT group. For 6/8 patients of the NT group, the basic causes of death were chagasic cardiopathy, and the final event was congestive heart failure. One patient died due to COVID-19, and the eighth died without medical assistance, probably due to cardiac sudden death, considering that he presented the mixed clinical form of CD. In the BZ-treated group, the only patient who died had moderate cardiac involvement on echocardiogram, and we don’t know the cause of death because he moved out of the municipality.

The mortality rate was estimated considering the total of 1,003 person-years (441 for NT and 562 for BZ-T) at risk during the follow-up-time. The mortality rates were 1.7/1,000 person-years (95% CI: 0.09-8.77) for BZ-T and 18.1/1,000 person-years (95% CI: 0.84-3.45) for NT (Table 6).

**Table 6:** Survival estimates for mortality of patients with chronic Chagas disease categorized as BZ-treated and NT upon 27-year follow-up.

BZ-Treatment	Number of Events	Overall Mortality by 100	Number of Person-years	Mortality Rate by 1,000 person-years	Adjusted HR*
				(95% CI)	(95% CI)
**No**	8	38.1	441	18.1 (0.84-3.45)	1.0 (reference)
**Yes**	1	4.8	562	1.7 (0.09-8.77)	0.09 (0.01-0.77)

*Adjusted by age and sex; HR = Hazard Ratio; CI = Confidence Interval.

**Fig 5:**
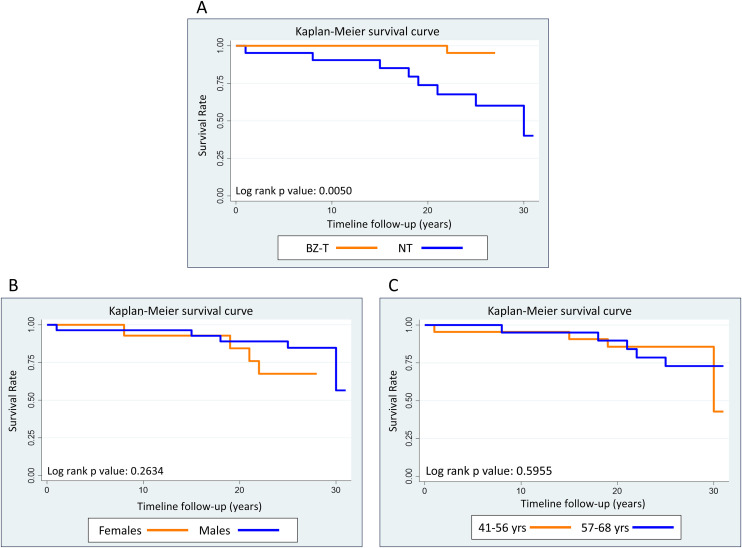
Overall analysis of the survival probability for death of patients with chronic CD categorized according to: (A) BZ-treatment [BZ-T and NT], (B) sex [males and females] and (C) age ranges [41-56 and 57-68 years old].

The overall survival probability for death was significantly higher (p = 0.0050) in BZ-T (95.0%) as compared to the NT group (40.0%) (Fig 5A). The survival probability curves did not demonstrate statistical differences between males and females (Fig 5B), as well as, between patients aged 41–56 and 57–68 years at 27-years (Fig 5C).

The Cox proportion hazard model showed that treatment emerged as a significant protective factor associated with a 91.0% reduction in the risk for death by CD, adjusted by sex and age in the final model (HR 0.09; 95% CI; 0.01-0.77). Neither sex nor age were independently associated with mortality risk, but both variables were retained in the final model (Table 6).

## Discussion

One of the primary challenges in the treatment of CD in Brazil is the limited availability and accessibility of therapeutic drugs within public health services. Although etiological treatment with BZ has demonstrated high parasitological cure rates during the acute phase [7], a significant proportion of patients, particularly those in the chronic phase, continue to find barrier to receiving treatment.

CD remains a neglected tropical disease, predominantly affecting rural areas, impoverished, and socially marginalized populations. These communities often experience substandard housing conditions, a lack of basic sanitation, limited health infrastructure, inadequate access to health education, and insufficient information [50]. Moreover, the lack of training among health professionals in diagnosing, clinically evaluating, and managing CD contributes to its underdiagnosis and undertreatment, thereby facilitating ongoing transmission and increasing the risk of progression to severe clinical forms [8]. The Second Brazilian Consensus on Chagas disease from 2015 [7] recommends etiological treatment for patients in the chronic phase with indeterminate, mild cardiac, or digestive clinical forms, aiming at preventing or delaying disease progression to more severe manifestations of the disease [11,18]. However, despite the complex pathogenesis and prolonged clinical course of CD, significant obstacles exist to precisely evaluating the efficacy of the etiological treatment. In particular, remains a challenge to establish clear evidence of the etiological treatment impact on parasitological cure and in the long-term clinical evolution of BZ-treated *versus* NT patients. Over time, several randomized studies have suggested either a lack of therapeutic efficacy or limited benefit of etiological treatment when administered during the chronic phase of CD. One example is the [51] study, which differs from several publications because only patients with the disease in the advanced stage of cardiac involvement were included. Previous studies have reported persistent infection, continued clinical progression, and no significant difference in primary outcomes such as mortality, when compared to the not treated after long-term follow-up. Conversely, a growing body of evidence supports the benefits of antiparasitic treatment in the late chronic phase, with studies documenting negative serology and parasitological cure following treatment [7,10,24,52–55].

These conflicting findings may be attributed to differences in therapeutic regimens, cure criteria, genetic and immunological diversity of parasite and host, age at treatment, variability in follow-up duration, and divergent methodologies employed for post-treatment evaluation. Additionally, a lack of awareness or reluctance among healthcare professionals to prescribe antiparasitic therapy during the different disease phases may further contribute to these divergences [8,14,20–22].

In light of these discrepancies and the pressing need for new data to support evidence-based practices, this study aimed to assess the long-term impact of etiological treatment in patients with chronic CD residing in Berilo municipality, Jequitinhonha Valley, MG, Brazil, a region historically endemic for CD with intense vector transmission prior decades. BZ-treated and NT patients were evaluated.

This longitudinal study focused on determining the real benefit of etiological treatment of CD through comparative evaluations conducted 9, 13 and 27-year follow-up. We assessed survival and death in BZ-treated patients and NT, using a historical cohort design with a follow-up duration unprecedented in the literature.

This cohort includes 42 patients divided into two equal groups, treated and NT, periodically evaluated at 9 years after treatment with 28 patients [41], 13 years after treatment with 29 patients [42], and 27 years after treatment. Although the NT group has been evaluated, it was not included in the 9-year follow-up study [41]; it was included in the 13-year [42] and 27-year follow-up assessments.

Diagnostic assessments included parasitological methods with different sensitivity and specificity, such as HC [44], PCR [45], and qPCR [47]; conventional serological tests (ELISA, IIF, IHA), and non-conventional serology (flow cytometry-based in the research of anti-live amastigotes and trypomastigote and fixed epimastigote antibodies - Chagas-Flow ATE,) [26], which allowed the establishment of three cure criteria. The outcome was determined based on the presence of patients in the assessments and mortality obtained through municipal health and official death certificates.

Demographic and epidemiological assessments revealed a predominance of females in both groups, likely reflecting higher health-seeking behavior among women. This aligns with findings from [56], which indicate a 1.7-fold greater risk of disease progression and mortality in males, possibly due to a lower frequency of diagnosis and medical follow-up.

Most participants resident in rural areas and engaged in subsistence farming, a profile consistent with CD historical association with socioeconomically marginalized, poorly housed rural populations; limited access to healthcare and vector surveillance before the 1990s may have contributed to disease persistence [43]. Disparities in healthcare access between urban and rural areas have been documented [57], emphasizing structural barriers faced by rural populations.

Parasitological results indicated that HC was negative in 90.5% of the patients, 9 and 13-year follow-up, and 100% in 27-year follow-up, respectively. In contrast, 52.4% of the Not-treated participants had positive HC at 9-year follow-up, but all were negative after 27-year follow-up.

These findings reinforce the effectiveness of BZ in achieving early parasitological clearance and are consistent with the studies [19,58,59] that demonstrated negative HC in the first 2–3 years after treatment, whereas the absence of treatment likely contributed to the persistence of infection during the same period in not-treated patients [13].

Despite these favorable outcomes, it is important to recognize the limitations of HC as a diagnostic method, particularly in the chronic phase of CD. Although its positive result unequivocally confirms the presence of viable parasites, the low sensitivity of this technique may lead to false-negative outcomes, especially when the parasitemia is low. Therefore, its limited sensitivity requires the complementary use of more sensitive molecular techniques to ensure a rigorous and more accurate evaluation of treatment efficacy [58].

Polymerase chain reaction (PCR) has emerged as a more sensitive tool for detecting *T. cruzi* DNA [60], and consequently the infection in treated patients. In the BZ-treated group, PCR analyses were negative in 23.8% at 9-years, 33.3% at 13-years, and 100% at 27-year post-treatment. On the other hand, only 14.3% in the NT group were PCR negative at 9 years after diagnosis, and total (100%) PCR negative after 27-year follow-up. These last results suggest a natural reduction in parasitemia, possibly driven by the host immune mechanism, as proposed [61].

The discrepancy between PCR and HC outcomes highlights PCR´s high sensitivity, although the potential for false-positive results due to residual k-DNA fragments must be considered. Quantifying parasitic load by qPCR could enhance the interpretation of PCR results and help distinguish between active infection and residual DNA. Although PCR can detect small quantities of k-DNA, often less than that contained in a single parasite, quantitative PCR (qPCR) could provide a more robust alternative for defining the genuine positivity and monitoring the therapeutic response.

Our data are consistent with the findings of [61–63], which reported a higher PCR positivity in the early years following treatment and a subsequent increase in negativity during the follow-up in treated and NT patients. In the study [61], a longitudinal monitoring of the parasite load in treated patients showed PCR positivity of 79% at diagnosis, which decrease to 29% and 24% in the initial years post-treatment, followed by a progressive increase in positivity, ranging from 30-69%, approximately 10 years after BZ-treatment, differently than the observed in treated children that showed seroconversion and more consistent negative parasitological tests. In contrast, our data demonstrated a progressive decline in PCR positivity over time, extending approximately 27 years of follow-up, a monitoring duration never reported in the literature. Differences between these findings may be partially explained by regional variations in *T. cruzi* genetics, with TcV predominant in the study region [61], and TcII being the most frequent lineage in our cohort [64].

In the present study, the quantitative molecular analyses were performed only at 27-year follow-up and revealed complete negativity in both BZ-treated and NT treated groups. Despite its high sensitivity and specificity [65], qPCR has limited diagnostic utility in chronic CD due to typically low parasitemia in this phase [37,60]. The publication [66] reported a 20% qPCR positive 3-year follow-up, which increased to 38.5% when combined with HC. However, given that only a minority of patients tested positive by qPCR at baseline in that publication, further investigations would be needed to determine its real value in monitoring parasite persistence or reduction over time post-treatment.

Our findings showing qPCR test negative in all patients evaluated by this technique after 27-year follow-up, corroborate this trend. It is also plausible that differences in *T. cruzi* genetic diversity and host immunological characteristics may influence parasitemia and diagnosis sensitivity [39,67]. Another relevant aspect is the virulence of the *T. cruzi* strains circulating in the studied area. Our publication [68] reported that most strains isolated from children in the study region exhibited low (6 out of 8 cases) or, at most, moderate levels of parasitemia, which may partially explain the difficulty in detecting circulating parasites in chronically infected patients. These findings highlight the potential influence of regional parasite biology on diagnostic performance and the interpretation of post-treatment parasitological outcomes [69].

Conventional serology assays (ELISA) and (IFI and IHA in some evaluations, showed persistent reactivity in all treated patients at 9 and 13-year follow-up, and 75% of seroconversion at 27-year follow-up. In contrast, 23.1% of the NT patients presented negative results in ELISA and non-conventional serology, what will be explored later. This delayed but progressive seroconversion post-treatment supports trypanocidal efficacy of BZ, corroborating the results of [15,70,71], as well as [41,42,72]. The [73] meta-analysis, suggested that the disappearance of anti-*T. cruzi* antibodies, when observed alongside negative parasitological and molecular test results, in chronically infected patients following treatment, may indicate trypanocidal effect in the long-term follow-up. Eliminating the parasite by treatment probably led to a gradual reduction in immunological stimulation, ultimately resulting in serological tests with low reactivity or not reactive, a trend not observed in patients who did not receive specific treatment.

Non-conventional serology using the Chagas-Flow ATE method revealed seroconversion of 90% in the treated group after 27-year follow-up, compared to 23.4% in the NT group. This method proved more effective in detecting parasitological cure than conventional ELISA, supporting its value in monitoring therapeutic efficacy [22]. Our findings corroborate those of [63,64], who conducted post-treatment monitoring in patients with chronic CD from the same municipalities using non-conventional serological methods such as Chagas-Flow ATE and FC-ALTA. Notably, [63] observed a 50% reduction in serological reactivity of FC-ALTA in treated children after 20-year follow-up, reinforcing the concept that long-term monitoring can capture gradual immunological changes with reduction of IgG1 anti-*T. cruzi* antibodies, indicative of therapeutic success. Therefore, the Chagas-Flow ATE method and similar flow cytometry-based assays allow more sensitive detection of IgG antibodies specifically associated with active *T. cruzi* infection [74,75], offering an advantage over conventional serology tests, in which antibody persistence of long duration and in low level does not necessarily reflect ongoing parasitemia [25,74].

This enhanced sensitivity makes these methods particularly promising for post-treatment monitoring in chronic CD, where conventional serology seroconversion may take decades in cases of parasitological cure. By targeting antibodies more directly linked with active infection, flow cytometry-based techniques may help distinguish between residual infection and successful parasite clearance, enabling a more accurate evaluation of therapeutic outcomes and potentially guiding clinical decision-making with greater confidence.

However, during the chronic phase, many BZ-treated or NT patients exhibit low or undetectable parasitemia, which complicates the direct detection of *T. cruzi*. In some patients, this fact is still allied to the absence of CD symptoms. In our study, the indirect detection of the infection through the presence of anti- *T. cruzi* antibodies by serological test have become essential, especially considering that even the highly sensitive qPCR failed to detect parasite k-DNA in blood samples. This finding suggests that the parasite may persist in tissue reservoirs without detectable circulating DNA, thereby sustaining a serological response despite negative molecular results. Thus, the combined use of parasitological and serological methods offers a more comprehensive and reliable evaluation of therapeutic outcomes.

Using distinct cure criteria, we evaluated therapeutic outcomes and observed varying levels of parasitological cure. Data from our analyses were interpreted using criteria referred as: classic, second and third alternative criteria. According to the classic cure criterion proposed by [7], no parasitological cure was observed in either the treated or untreated groups during the early follow-up years (9 and 13-years). However, after 27 years, 75% of treated patients met the classic cure criteria. Interestingly, 23.1% of the NT patients also met the same criteria, suggesting the possibility of spontaneous or auto-cure, which warrants further investigation. The second criterion, which included the detection of anti-amastigote antibodies, known to anticipate seroconversion earlier than conventional serology ELISA, identified a higher cure rate of 95% among treated patients. In contrast, the third cure criterion, which employed highly sensitive qPCR, did not identify any new cases of parasitological cure. Probably, if we had performed other evaluations of the patients in the interval between the present evaluation and the previous one [42], we could have discovered before negative results in the Chagas Flow- ATE test.

Our findings clearly demonstrate that the use of the non-conventional Chagas-Flow ATE method is a valuable tool for monitoring therapeutic response and assessing parasitological cure in the chronic phase of CD. The classic cure criterion [7] underestimate treatment success due to its dependence on late seroconversion of the conventional serological tests, which can take decades to occur in chronically infected patients.

The clinical evolution of the patients of the cohort here evaluated is a subject of another publication supporting the results presented here. Our data contributed to a more nuanced understanding of post-treatment outcomes and highlights the importance of integrating multiple diagnostic strategies for a robust assessment of cure.

CD continues to be a serious public health problem, with high mortality rates in several Latin American countries. Study [76] demonstrated, based on a nationwide population-based ecological study, mortality from CD between 2000 and 2019. During this period, 22,663,092 total deaths were recorded, with 122,291 due to Chagas disease, 94,788 (77.5%) as the underlying cause and 27,503 (22.05%) as associated cause. Mortality and overall survival in our study were assessed 27-year follow-up. The observed overall mortality was lower among BZ-T patients compared to NT patients. Our data demonstrated 91.0% reduction in the risk of overall death (p = 0.028) in BZ-treated compared to NT patients. Furthermore, the survival estimate for death was higher in BZ-treated group compared to the NT group (p = 0.006). These findings are discordant with those found in comparative studies with treated and untreated patients at different follow-up periods. The work [77] reported no differences in mortality rate between treated and untreated patients after 5-year follow-up, and [55] did not find an association between BZ-treatment and death after ~33-year follow-up (median 15 years).

It is reasonable to suppose that the etiological treatment can minimize the risk of more serious clinical complications, such as cardiac and/or digestive abnormalities, which consequently reduces the risk of death from complications inherent of CD. We believe that a long-term clinical follow-up can provide a better characterization of disease progression in untreated patients and further demonstrate the beneficial impact of BZ-treatment. It is suggestive that BZ-treatment reduce the parasitemia with consequent lower clinical evolution, which could be associated with a lower risk of death when compared to the NT treated control group [52,54]. The publication [54] demonstrated that patients previously treated with BZ presented reduced parasitemia, lower prevalence of severe heart disease, and lower mortality when compared to the not-treated control group after 2-year follow-up. Our study confirmed the importance of the BZ-treatment in the chronic phase of CD, pointing out a lower risk of death amongst BZ-treated patients, as previously reported by several authors [11,24,54,55].

Although this study has limitations, mainly regarding the low number of patients in the cohort, the long interval between the last two patients’ evaluations, which together represent possible bias, in addition to the difficulty in patients’ adherence to the study. Given that clinical differences between the groups were not evaluated, selection bias may have been introduced. Nonetheless, matching by age and sex likely reduced imbalances in baseline characteristics at cohort entry. Loss to follow-up between treatment and our first evaluation was primarily due to patients’ migration to other areas, which may have reduced the precision of the estimates results. Despite these limitations, a major strength of this study is its long clinical follow-up period, which provides a more robust assessment of disease progression.

We believe that our results will inspire the medical and scientific community to consider the etiological treatment for patients at chronic CD, considering the benefits reposted in the present investigation. Furthermore, the Health Ministerial Ordinance No. 264, of February 17, 2020, assigns our Unified Health System the responsibility of diagnosing the infection, evaluating and clinically assisting diagnosed cases, and offering etiological treatment according to the II Brazilian Consensus on Chagas Disease, which unfortunately has not been done usually.

## Conclusion

In conclusion, our results demonstrate that BZ-treatment can lead to parasitological cure in a significant proportion of patients with chronic CD, especially when monitored over a long-term follow-up. Therefore, BZ-treatment should be encouraged even in the chronic phase of CD. The integrative analysis of several laboratory methods with different principles, including HC, PCR, qPCR, along with conventional and non-conventional serological tests, proved to be essential for a more sensitive and comprehensive evaluation of therapeutic response. In particular, we highlight the utility of flow cytometry-based serology (Chagas-Flow ATE), which showed greater sensitivity in detecting antibodies associated with active infection and contributed to a more accurate estimation of cure. These findings challenge the exclusive reliance on the classical cure criterion, which depends on delayed seroconversion of the conventional serology, and support the importance of combined and long-term diagnostic strategies for properly assessing the BZ-treatment effectiveness in endemic settings. In addition, we demonstrated a significantly higher overall survival in the BZ-treated group as compared to the NT patients, suggesting a lower risk of death amongst BZ-treated patients in the chronic phase of CD.

## Supporting information

S1 Table Overall reactivity of samples from BZ-treated chronic CD patients at 27-year follow-up in conventional and non-conventional serology. ID = Identification number; Males; F = Females; N = Negative results; P = Positive results; * = Death.
(DOCX)

S2 Table Overall reactivity of samples from BZ not-treated chronic CD patients at 27-year follow-up in conventional and non-conventional serology. ID = Identification number; Males; F = Females; N = Negative results; P = Positive results; * = Death.
(DOCX)
