# Supplementary material for: Long-term outcomes of benznidazole treatment in chronic Chagas disease: A 27-year cohort study of parasitological cure and death in the Jequitinhonha Valley, Brazil
Source: PLoS Negl Trop Dis. 2025 Nov 20;19(11):e0013619. doi: 10.1371/journal.pntd.0013619 (PMC12633969; doi:10.1371/journal.pntd.0013619)
Supplement: S1 Table — ID = Identification number; Males; F = Females; N = Negative results; P = Positive results; * = Death. (DOCX) [file pntd.0013619.s001.docx]

| **ID** | **Demographic data** | |  | **Serological methods** | | | | | | | | |  |
| --- | --- | --- | --- | --- | --- | --- | --- | --- | --- | --- | --- | --- | --- |
|  |  |  |  | **Conventional serology- ELISA** | |  | **Non-conventional serology– Chagas-Flow ATE** | | | | | | |
|  | **Sex** | **Age** |  |  |  |  | **Amastigote** | | **Trypomastigote** | | **Epimastigote** | | |
|  |  |  |  | **Cut-off (0.317)** | **Anti-*T. cruz*** |  | **Cut-off (40%)** | **Anti-AMA** | **Cut-off (20%)** | **Anti-TRYPO** | **Cut-off (20%)** | **Anti-EPI** | |
| **447** | M | 50 |  | 0.356 | P |  | 25.4 % | N | 26.9 % | P | 39.1 % | P | |
| **543** | M | 68 |  | * | * |  | * | * | * | * | * | * | |
| **772** | F | 62 |  | 0.338 | P |  | 8.6 % | N | 13.1 % | N | 74.9 % | P | |
| **1007** | F | 49 |  | 0.270 | N |  | 2.6 % | N | 2.7 % | N | 3.0 % | N | |
| **1091** | M | 53 |  | 0.276 | N |  | 3.43 % | N | 10.7 % | N | 7.6 % | N | |
| **1092** | F | 56 |  | 0.356 | P |  | 7.4 % | N | 7.5 % | N | 6.3 % | N | |
| **1095** | M | 55 |  | 0.286 | N |  | 2.51 % | N | 10.0 % | N | 7.6 % | N | |
| **1096** | M | 58 |  | 0.265 | N |  | 2.21 % | N | 3.3 % | N | 3.84 % | N | |
| **1097** | M | 59 |  | 0.372 | P |  | 97.3 % | P | 99.2 % | P | 92.4 % | P | |
| **1098** | F | 63 |  | 0.283 | N |  | 20.0 % | N | 15.5 % | N | 58.6 % | P | |
| **1100** | F | 61 |  | 0.444 | P |  | 82.7 % | P | 87.7 % | P | 96.3 % | P | |
| **1102** | F | 56 |  | 0.274 | N |  | 3.25 % | N | 4.8 % | N | 5.5 % | N | |
| **1103** | F | 56 |  | 0.255 | N |  | 4.25 % | N | 5.6 % | N | 12.9 % | N | |
| **1104** | F | 53 |  | 0.298 | N |  | 22.6 % | N | 41.7 % | P | 19.4 % | N | |
| **1105** | F | 60 |  | 0.299 | N |  | 8.36 % | N | 9.6 % | N | 20.0 % | N | |
| **1108** | F | 53 |  | 0.274 | N |  | 10.8 % | N | 9.8 % | N | 8.1 % | N | |
| **1109** | F | 59 |  | 0.245 | N |  | 2.83 % | N | 3.4 % | N | 8.3 % | N | |
| **1110** | F | 56 |  | 0.314 | N |  | 33.3 % | N | 93.4 % | P | 74.1 % | P | |
| **1111** | M | 57 |  | 0.292 | N |  | 3.2 % | N | 6.2 % | N | 5.9 % | N | |
| **1112** | F | 63 |  | 0.315 | N |  | 2.71 % | N | 4.1 % | N | 5.3 % | N | |
| **1113** | F | 41 |  | 0.286 | N |  | 2.63 % | N | 4.3 % | N | 7.6 % | N | |
| **(%)** | **-** | **-** |  | **-** | **75.0** |  | **-** | **90.0** | **-** | **75.0** | **-** | **70.0** | |

**S1 Table.** Overall reactivity of samples from BZ-treated chronic CD patients at 27-year follow-up in conventional and non-conventional serology

ID = Identification number; Males; F = Females; N = Negative results; P = Positive results; * = Death.
