# Supplementary material for: Long-term outcomes of benznidazole treatment in chronic Chagas disease: A 27-year cohort study of parasitological cure and death in the Jequitinhonha Valley, Brazil
Source: PLoS Negl Trop Dis. 2025 Nov 20;19(11):e0013619. doi: 10.1371/journal.pntd.0013619 (PMC12633969; doi:10.1371/journal.pntd.0013619)
Supplement: S2 Table — ID = Identification number; Males; F = Females; N = Negative results; P = Positive results; * = Death. (DOCX) [file pntd.0013619.s002.docx]

| **ID** | **Demographic data** | |  | **Serological methods** | | | | | | | | |  |
| --- | --- | --- | --- | --- | --- | --- | --- | --- | --- | --- | --- | --- | --- |
|  |  |  |  | **Conventional serology- ELISA** | |  | **Non-conventional serology– Chagas-Flow ATE** | | | | | | |
|  | **Sex** | **Age** |  |  |  |  | **Amastigote** | | **Trypomastigote** | | **Epimastigote** | | |
|  |  |  |  | **Cut-off (0.317)** | **Anti-*T. cruz*** |  | **Cut-off (40%)** | **Anti-AMA** | **Cut-off (20%)** | **Anti-TRYPO** | **Cut-off (20%)** | **Anti-EPI** | |
| **125** | F | 56 |  | 0.308 | N |  | 23.8 | N | 21.0 | P | 49.7 | P | |
| **389** | F | 64 |  | 0.379 | P |  | 49.8 | P | 83.9 | P | 97.3 | P | |
| **403** | M | 45 |  | 0.414 | P |  | 55.3 | P | 84.2 | P | 97.2 | P | |
| **415** | F | 56 |  | 0.361 | P |  | 62.1 | P | 62.0 | P | 89.6 | P | |
| **438** | F | 50 |  | * | * |  | * | * | * | * | * | * | |
| **441** | F | 60 |  | 0.305 | N |  | 24.9 | N | 46.0 | P | 13.9 | N | |
| **443** | M | 57 |  | 0.382 | P |  | 52.4 | P | 67.4 | P | 87.2 | P | |
| **493** | M | 58 |  | 0.405 | P |  | 97.4 | P | 96.2 | P | 89.8 | P | |
| **551** | F | 55 |  | 0.391 | P |  | 67.2 | P | 74.7 | P | 93.6 | P | |
| **562** | M | 59 |  | * | * |  | * | * | * | * | * | * | |
| **640** | F | 61 |  | * | * |  | * | * | * | * | * | * | |
| **798** | F | 56 |  | * | * |  | * | * | * | * | * | * | |
| **830** | F | 54 |  | * | * |  | * | * | * | * | * | * | |
| **894** | F | 54 |  | 0.334 | P |  | 66.3 | P | 98.4 | P | 69.2 | P | |
| **929** | M | 55 |  | * | * |  | * | * | * | * | * | * | |
| **1365** | F | 63 |  | * | * |  | * | * | * | * | * | * | |
| **1422** | F | 58 |  | 0.383 | P |  | 61.5 | P | 95.7 | P | 86.5 | P | |
| **2151** | M | 68 |  | * | * |  | * | * | * | * | * | * | |
| **2438** | F | 46 |  | 0.231 | N |  | 3.1 | N | 3.0 | N | 2.4 | N | |
| **2440** | M | 53 |  | 0.348 | P |  | 45.5 | P | 56.3 | P | 94.4 | P | |
| **2464** | F | 61 |  | 0.459 | P |  | 97.6 | P | 99.0 | P | 7.6 | P | |
| **(%)** | **-** | **-** |  | **-** | **23.1** |  | **-** | **23.1** | **-** | **7.7** | **-** | **15.4** | |

**Table S2.** Overall reactivity of samples from NT chronic CD patients at 27-year follow-up in conventional and non-conventional serology

ID = Identification number; Males; F = Females; N = Negative results; P = Positive results; * = Death.
